# The choice of 16S rRNA gene sequence analysis impacted characterization of highly variable surface microbiota in dairy processing environments

**DOI:** 10.1128/msystems.00620-24

**Published:** 2024-10-21

**Authors:** Sarah E. Daly, Jingzhang Feng, Devin Daeschel, Jasna Kovac, Abigail B. Snyder

**Affiliations:** 1 Department of Food Science, Cornell University, Ithaca, New York, USA; 2 Department of Food Science, Pennsylvania State University, University Park, Pennsylvania, USA; Kobenhavns Universitet, Frederiksberg C, Denmark

**Keywords:** dairy processing environments, bioinformatics, food safety, amplicon sequencing, microbial ecology

## Abstract

Accurate knowledge of the microbiota collected from surfaces in food processing environments is important for food quality and safety. This study assessed discrepancies in taxonomic composition and alpha and beta diversity values generated from eight different bioinformatic workflows for the analysis of 16S rRNA gene sequences extracted from the microbiota collected from surfaces in dairy processing environments. We found that the microbiota collected from environmental surfaces varied widely in density (0–9.09 log_10_ CFU/cm^2^) and Shannon alpha diversity (0.01–3.40). Consequently, depending on the sequence analysis method used, characterization of low-abundance genera (i.e., below 1% relative abundance) and the number of genera identified (114–173 genera) varied considerably. Some low-abundance genera, including *Listeria*, varied between the amplicon sequence variant (ASV) and operational taxonomic unit (OTU) methods. Centered log-ratio transformation inflated alpha and beta diversity values compared to rarefaction. Furthermore, the ASV method also inflated alpha and beta diversity values compared to the OTU method (*P* < 0.05). Therefore, for sparse, uneven, low-density data sets, the OTU method and rarefaction are better for taxonomic and ecological characterization of surface microbiota.

**IMPORTANCE:**

Culture-dependent environmental monitoring programs are used by the food industry to identify foodborne pathogens and spoilage biota on surfaces in food processing environments. The use of culture-independent 16S rRNA amplicon sequencing to characterize this surface microbiota has been proposed as a tool to enhance environmental monitoring. However, there is no consensus on the most suitable bioinformatic analyses to accurately capture the diverse levels and types of bacteria on surfaces in food processing environments. Here, we quantify the impact of different bioinformatic analyses on the results and interpretation of 16S rRNA amplicon sequences collected from three cultured dairy facilities in New York State. This study provides guidance for the selection of appropriate 16S rRNA analysis procedures for studying environmental microbiota in dairy processing environments.

## INTRODUCTION

Environmental monitoring activities involve the collection of surface swabs from built environments, such as food processing plants, followed by the evaluation of pathogens, spoilage biota, or indicator organisms collected on the swabs (1, 2). The food industry uses these programs to “seek and destroy” reservoirs of pathogenic or spoilage biota before cross-contamination into the product can occur. The most common use of environmental monitoring programs is *Listeria* spp. detection, but quantification of indicators such as total plate count or Enterobacteriaceae may be used to monitor hygiene. A number of recent studies have applied culture-independent amplicon sequencing to environmental monitoring. Amplicon sequencing can identify novel microbial indicators (3), map potential microbial relationships (4), and link environmental influences to microbial characteristics (5). For example, amplicon sequencing has been used to identify patterns in surface microbiota associated with the presence of *Listeria* spp. (6, 7) and to identify changes in the surface microbiota across facilities or swab sites (8–11). Amplicon sequencing has also been used to quantify differences in diversity or identity of surface microbiota, which can then be linked with different hygienic conditions (12–16). The aim of culture-independent approaches to environmental monitoring is to develop more sensitive, proactive measures that do not rely exclusively on the detection of low-occurrence pathogens.

One challenge in the application of amplicon sequencing within environmental monitoring programs is the significant variation among swab site locations that likely impact the density (i.e., number of cells per unit area) and diversity (i.e., the number and abundance of unique taxa) of microbiota. We suspect that this, in turn, impacts downstream taxonomic and ecological outputs depending on the set of bioinformatic analyses deployed. These multi-step bioinformatic analyses include quality-checking, trimming, aggregation, normalization, and alignment of the sequence data (17, 18). The taxonomic and ecological characteristics (i.e., relative abundance, alpha, beta diversity) can be obtained from these analyses and are used to draw conclusions from these data sets (19, 20). However, differences in taxonomic and ecological outputs from the same data set using different bioinformatic analyses have been reported (21–23). For example, the selection of amplicon sequence variants (ASVs) versus operational taxonomic units (OTUs) for sequence aggregation has caused discrepancies in outcomes for community composition (24) and diversity values (25, 26).

There have been few investigations quantifying the effects of these different sequence analyses on taxonomic and ecological characterizations of microbiota from dairy processing environments. This is particularly true for analyses performed on samples collected from surfaces in food processing environments which possess many unique attributes. Therefore, this study aims to determine (i) the impact of sample attributes (i.e., microbial density) on taxonomic and diversity values derived from different combinations of bioinformatic analyses, namely sequence aggregation, sequencing depth, and normalization, and (ii) the most appropriate set of bioinformatic analyses for future research applying amplicon sequencing to surface swabs collected from food processing environments. Overall, this study will better quantify the impact of different sample attributes and bioinformatic analyses on the characterization of microbiota collected from surfaces in food processing environments.

## MATERIALS AND METHODS

### Sample collection and processing

Environmental surface swabs were collected from three different cultured dairy facilities in New York State. Facility 1 was 16,537 m^2^ and produced cheese, sour cream, and yogurt products. At the time of sampling, there were approximately 195 personnel employed. Facilities 2 (3,902 m^2^) and 3 (11,286 m^2^) both primarily produced yogurt products. At the time of sampling, Facility 2 had approximately 40 personnel employed. Facility 3 had approximately 100 personnel employed.

A total of 636 environmental surface samples were collected from July 2022 to May 2023. Each facility was sampled a total of four times (i.e., summer, fall, winter, and spring). Surface swab samples were collected from zone 2, 3, and 4 areas within each facility. In subsequent sampling trips at each facility, the same or similar surfaces were swabbed as in the previous visits.

Surfaces were swabbed with a sponge (Neogen 3M, Neogen, Lansing, MI, USA) or a cotton swab premoistened with 10 mL Dey/Engley (D/E) D/E buffer. Sponges were used to collect samples from large areas of flat surfaces (e.g., surface of walls, floor), whereas cotton swabs were used when sampling from surfaces with small, recessed spaces (e.g., small junction of the wall). Each surface was swabbed 10 times vertically and 10 times horizontally over a 30.5 × 30.5-cm surface area. The cotton swabs or sponges were immediately stored and sealed into sterile bags (Whirl-Pak, Pleasant Prairie, WI, USA). The cotton swabs and sponges were stored in a cooler and processed within 24 hours of collection.

Each sample bag received 40 mL of phosphate-buffered saline (PBS), representing a fivefold dilution. The liquid within each sample bag was homogenized by stomaching the bag for 2 minutes at high speed. Aliquots (5 mL) for the aerobic plate count (APC) were removed from the sample bag of homogenized liquid. Serial dilutions (10^0^, 10^2^, 10^4^) of the aliquot were prepared using PBS. Then, 1 mL of each diluted suspension was plated on a 3M (Neogen 3M, Neogen, Lansing, MI, USA) APC Petrifilm to enumerate the mesophilic aerobic species. The Petrifilms were incubated at 35°C for 24 hours. Colonies were counted on plates that contained between 1 and 1,000 colonies and were expressed as log_10_ CFU/sponge.

### Metadata collection

**Fig 1:**
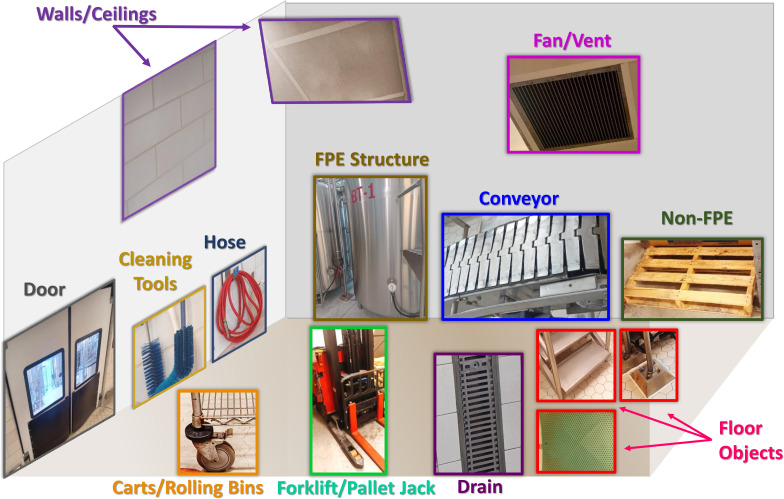
A schematic illustrating the 12 object types swabbed in a dairy processing environment.

During sample collection, we recorded metadata about the swabbed surfaces from each environmental site according to Feng et al. (2) (Tables S1 and S3). The environmental sites were categorized into 12 object types (floor, carts/rolling bins, forklifts/pallet jacks, fans/vents, hose, walls/ceilings, doors, conveyors, drains, cleaning tools, and non-food processing equipment [non-FPE] structures, and FPE structures) (Fig. 1; Table S2).

### DNA extraction and sequencing

The DNA was extracted from the homogenized samples using the Qiagen DNeasy PowerSoil DNA extraction kit (Qiagen, Germantown, MD, USA) with minor modifications of the manufacturer’s instructions (Qiagen, 2017). A 45-mL aliquot from each of the processed samples was centrifuged at 6,500 × *g* at 4°C for 20 minutes. The pellet was then transferred to the Powerbeads tube. Then, 800 µL of the CD1 buffer was added to the Powerbeads tube and vortexed (Vortex Genie) at maximum speed for 25 minutes. Lysates were cleaned up according to the manufacturer’s instructions. The concentration of DNA in the lysate was measured using a Qubit fluorometer. Samples with DNA concentrations lower than the detection limit of Qubit (i.e., 50 ng/mL) were removed. In total, 94 out of 643 (14.6%) samples were discarded due to a low DNA concentration.

The amplicon library was prepared by amplifying the 16S V3-V4 region, which is the standard region for amplification used in the standard Illumina 16S sequencing protocol (27). Amplicons were barcoded with unique index adaptors through PCR (Novogene, China). Libraries of barcoded amplicons were pooled in equal volumes and sequenced using the NovaSeq PE250 instrument and 500 cycle sequencing kit with a target of 100 kbp per sample library. Demultiplexed reads were filtered, and low-quality reads were removed in QIIME2 (v. qiime-2022.2). Paired-end reads were then trimmed if below a quality score of 20, denoised, and chimeras were removed.

### Sequence analysis workflows

**Fig 2:**
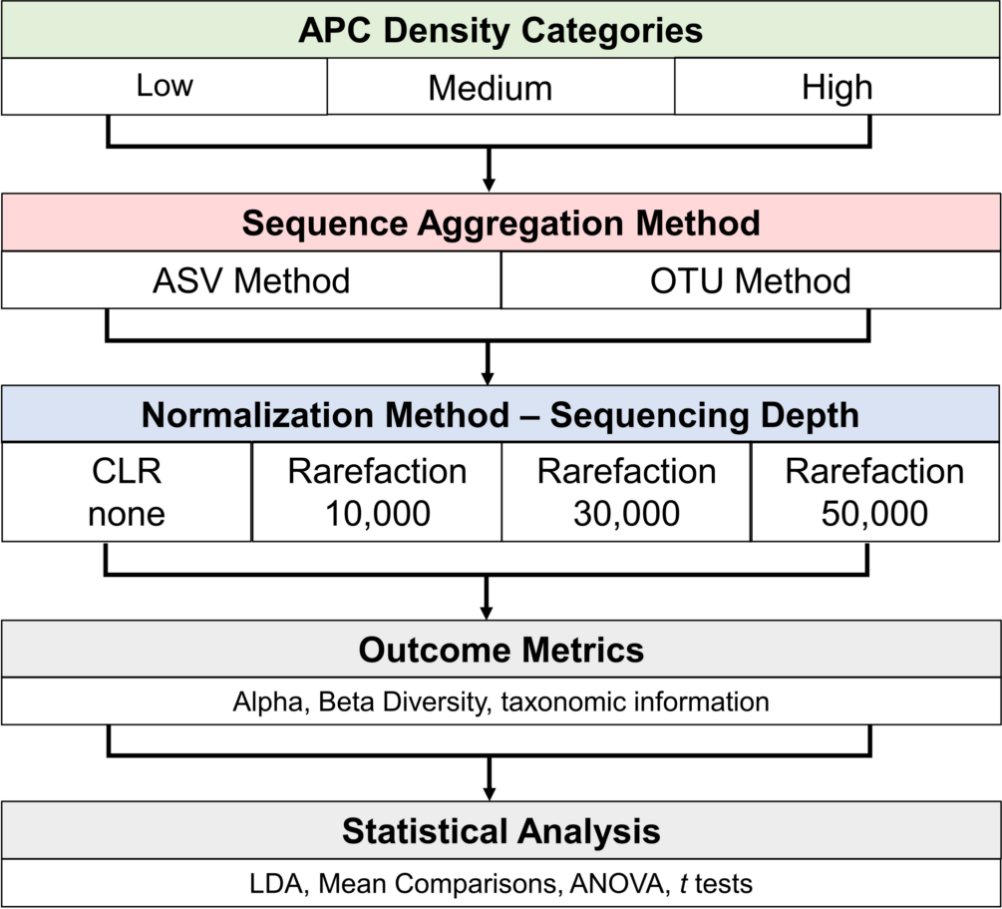
Overview of bioinformatic analyses. Samples from each of the three density categories underwent two different sequence aggregation methods and four types of normalization methods. Alpha and beta diversity and taxonomic information were collected from each pipeline and underwent statistical analysis. CLR, centered log-ratio; LDA, linear discriminant analysis; ANOVA, analysis of variance.

Sequences in each density category were analyzed in workflows containing different sequence aggregation or normalization methods as outlined in Fig. 2.

### APC density categories

Each environmental surface sample was binned into one of three microbial density categories, “low,” “medium,” or “high” (Table S1) based on its APC. “Low density” consisted of APC equal to or below 3 log_10_ CFU/sponge. “Medium density” consisted of APC between 3 and 5 log_10_ CFU/sponge. “High density” consisted of APC greater than or equal to 5 log_10_ CFU/sponge. To facilitate comparisons among microbial density categories, 100 samples were randomly selected from each density category and used for analysis

### Sequence aggregation method

The samples from each density category underwent (i) *de novo* clustering into OTUs at 99% similarity using vsearch (28) and (ii) generation of exact ASVs in DADA2 (29). A naïve Bayes classifier was trained using GreenGenes 99% phylogenetic tree v. 13.8 (30) reference sequences to classify the ASVs and OTUs.

### Normalization method sequencing depth

Each ASV and OTU table underwent two different normalization methods: (i) rarefaction and (ii) centered log-ratio (CLR) transformation using the phyloseq package (31) in RStudio (v. 2022-12-03) (32). In our study, rarefaction was conducted at three different sequencing depths (10,000, 30,000, and 50,000 reads). Fifty thousand was selected as the upper limit of sequencing depth based on viewing rarefaction curves of the data.

### Diversity analyses and calculations

Several outcome metrics were used to compare performances among different sequence analyses (Fig. 2). Alpha diversity was calculated using the Shannon, Chao1, and Pielou indices using the plot_richness function in the microbiomeutilities package (33) in RStudio. The Chao1 index captures community richness (number of species), the Pielou index captures community evenness (representation of species), and the Shannon index accounts for both species richness and evenness. Beta diversity was calculated using the Aitchison index in the betadispr function in the vegan package (34) in RStudio.

For each sequence analysis workflow, the total number of unique genera was reported. For workflows containing rarefaction, the number of genera for three categories of relative abundances (above 1%, between 1% and 0.01%, and below 0.01%) was reported. The percentage of zeros in the ASV and OTU tables (i.e., sparsity) was calculated for each sequence analysis workflow. The microbiota size (total number of bacteria) was determined by the plot_core function in the microbiome package (35) in RStudio. Lists of genera that did not overlap between the ASV and OTU methods were generated.

### Statistical analysis

Paired *t* tests were performed on alpha and beta diversity values and taxonomic units (ASVs versus OTUs) generated for individual environmental samples before normalization. *χ*^2^ analysis was performed using the chisq.test function in the stats package (32) in Rstudio to assess the correlation of residuals between object type categories and APC density categories. The impact of different sequence analyses on the outcome metrics (i.e., alpha and beta diversities, taxonomic composition) was determined using several statistical analyses in Rstudio. Tukey’s least means comparison was used to assess statistical differences between ASV-based and OTU-based methods for alpha and beta diversity values using the lsmeans function (36) in RStudio. Analysis of variance of non-overlapping genera was performed in the car package (37). Linear discriminant analysis (LDA) was performed using the lda function from the MASS package (38) in Rstudio to visualize the effect of a normalization method on alpha and beta diversity values. *P* values below 0.05 were considered significant.

## RESULTS AND DISCUSSION

### The microbiota collected from surfaces in dairy processing environments were characterized by variable aerobic plate counts, low-abundance genera, and low alpha diversity values

**Fig 3:**
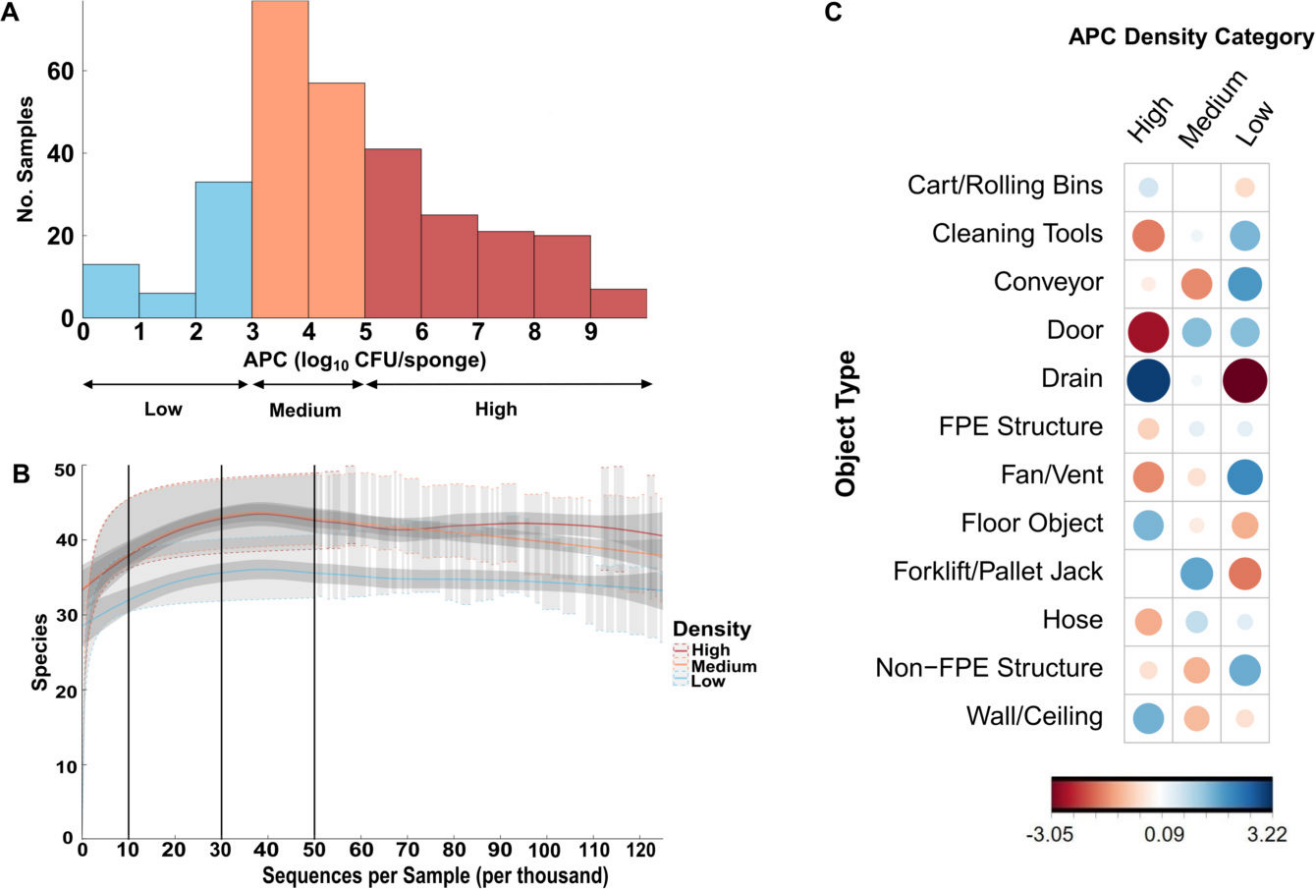
The 300 environmental samples were characterized by (**A**) the distribution of APC concentration, (**B**) the average number of sequencing reads in each density category, and (**C**) the *χ*^2^ correlation of residuals between object type and APC density category (*P* = 3.609e−06). Blue circles represent a positive association, and red circles represent a negative correlation. The diameter and color of the circle correspond to the strength of the correlation. The larger and darker the circle, the stronger the correlation.

There was a wide range of microbial densities among object types within the dairy processing environments. The aerobic plate count of environmental surface samples ranged from 0 to 9.09 log_10_ CFU/sponge (Fig. 3A; Table S1). The aerobic plate count category (i.e., high, medium, and low density) and object type were significantly correlated (*P* = 3.609e−06) (Fig. 3C). Specifically, drains, floor objects, and walls/ceilings were positively correlated with high aerobic plate counts (>5 log_10_ CFU/sponge). Conveyor belts and plant equipment, not directly used for food processing (non-FPE), were positively correlated with low aerobic plate counts (<3 log_10_ CFU/sponge) (Fig. 3C). Some object types, like hoses, FPE structures, and carts/rolling bins, were not strongly associated with any APC density category.

Each “object type” encompassed different sanitation regimes and exposure to various product materials. Some sites (i.e., conveyor belts) were regularly cleaned, whereas others, such as walls and ceilings, were cleaned at a much lower frequency (Table S1). Furthermore, most drains were continuously exposed to moisture and food products, while fans/vents were rarely if ever exposed to food residues or wet sanitation (Table S1). Individual sites within food processing environments can demonstrate large variability in APC based on sanitation regimes, object structure, and product exposure. For example, environmental monitoring of a commercial dairy facility found that concentrations of bacteria varied substantially by type of surface and their locations within different areas of the facility (39).

Furthermore, there may be a temporal component which affects APC on surfaces. The aerobic plate count at individual swabbing sites sometimes varied at different sampling dates. Of the 300 random samples, there were 150 unique environmental sites swabbed. Of those 150 sites, 98 had multiple sampling dates, i.e., the same surface was swabbed over multiple seasons (Table S1). In this study, 60% of repeated measurements of the same environmental surface were classified with at least one different microbial load (low, medium, or high aerobic plate count) during different seasons (Table S1). Further study is needed to examine seasonal effects on APC values in environmental monitoring.

**Fig 4:**
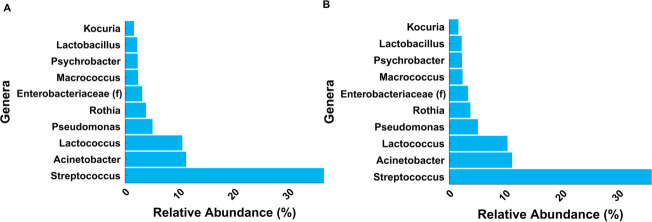
The 10 most abundant genera for all 300 environmental surface samples, as determined using (**A**) the ASV analysis method and (**B**) OTU analysis method. “(f)” indicates family.

The alpha diversity values provided insight into the microbial composition of these environmental surface samples. The alpha diversity varied substantially per sample ranging from 0.01 to 3.40 (Shannon diversity), 4 to 137 (Chao1 diversity), and 0.01 to 0.78 (Pielou diversity) (Table S1). These ranges were reflective of the variation of surface conditions through the facility. In one study, the diversity values for environmental surfaces in a cheese processing facility demonstrated considerable variability among different surface types with Chao1 values ranging from 50.63 to 155.50 and Shannon values ranging from 1.94 to 4.60 (40). Notably, the alpha diversity values for several samples in our study were quite low, with most samples below 50 for Chao1 diversity and 2 for Shannon diversity (Table S1). The evenness for some samples was very low with Pielou values as low as 0.01 (Table S1). The low evenness of many samples was most likely due to the overrepresentation of certain genera among the surface microbiota. The genera *Streptococcus*, *Lactococcus*, *Pseudomonas*, and *Acinetobacter* accounted for 63% of relative abundance across all environmental surface samples (Fig. 4). Low-abundance genera (<1% relative abundance) were prevalent (>150 genera) (Table 3) in environmental surface samples with both low and high aerobic plate counts. Microbiota found on the surfaces of food processing environments often have a core group of frequent and highly abundant taxa, while low-abundance taxa are the largest proportion of microbiota (8, 41–43). The core genera of *Streptococcus*, *Lactococcus*, *Pseudomonas*, and *Acinetobacter* found in this study are highly consistent with other studies of bacteria identified on surfaces in dairy processing environments (8, 10, 40).

These results demonstrate that surfaces in dairy processing environments commonly have highly variable aerobic plate counts and alpha diversity values. Furthermore, the microbiota on these surfaces are dominated by a few core genera but also consist of many low-abundance genera (i.e., are highly uneven). These attributes require specialized consideration of sequence analysis to adequately analyze the sequences for downstream interpretation.

### Differences in the ASV and OTU aggregation methods impacted characterization of low-abundance microbiota

The number of ASVs generated from the individual environmental surface samples (before normalization) was significantly higher (*P* < 0.001) than the number of OTUs generated (Tables S1 and S6). The ASV method also generated significantly higher alpha and beta diversity values (*P* < 0.001) compared to the OTU method (Table S6). Moreover, there were significant differences (*P* < 0.05) between the ASV and OTU methods for all the diversity indices of rarefied samples regardless of the sample density or sequencing depth (Tables 1 and 2). The ASV method generated a higher microbiota size (no. bacteria) than the OTU method (Table 3). Despite these differences in taxonomic unit generation, the OTU and ASV methods were consistent in the classification of the most abundant genera (>1% relative abundance) (Fig. 4). However, the ASV method identified fewer genera than the OTU method (Table 3). The number of genera identified below 0.1% relative abundance varied widely between sequence analyses (Table 3). There were several non-overlapping genera identified among different sequence aggregation methods (Table 4). Non-overlapping genera were somewhat more common in low-density samples (Table S4). In this study, the number and identity of low-abundance genera differed based on the sequence aggregation method. Notably, low-density samples analyzed using the pipelines applying the ASV method had very low relative abundances of the genus *Listeria* (Table S5). The genus *Listeria* fell below 0.1% relative abundance in the ASV method, which is a suggested cutoff in taxa filtering during bioinformatic analysis (44). Thus, *Listeria* sequences may be filtered out under this schema, which is a drawback as *Listeria monocytogenes* is the pathogen of concern associated with cheese and dairy processing environments (45). Other non-overlapping genera between the ASV and OTU methods included *Enterobacter*, *Flavobacterium*, and *Rahnella* (Table S5). These genera are relevant to food quality. For the analysis of microbiota collected from surfaces in food processing environments, the differences between sequence aggregation methods are important to consider, given the sparsity of the data set, and the large proportion of low-abundance genera. Therefore, the sequence aggregation method can impact alpha and beta diversity results when there are large proportions of rare taxa.

**TABLE 1:** Pipelines containing the ASV sequence aggregation method resulted in significantly higher diversity values for all rarefied data sets regardless of the sample density category

Normalization	Diversity index	Density category	Mean, ASV/OTU	CV^*a*^ (%), ASV/OTU	*P* value, mean contrast (ASV vs OTU)
Rarefaction	Shannon	High	1.68/1.38	43/48	<0.0001
		Medium	1.51/1.25	47/57	<0.0001
		Low	1.41/1.20	52/57	<0.0001
	Chao1	High	43.5/36.9	59/64	0.0197
		Medium	44.13/39.96	54/63	0.0197
		Low	36.1/36.2	59/63	0.0197
	Pielou	High	0.46/0.40	33/39	<0.0001
		Medium	0.41/0.35	38/46	<0.0001
		Low	0.40/0.34	46/51	<0.0001
	Aitchison	High	23.9/21.0	28/28	<0.0001
		Medium	23.03/20.49	30/34	<0.0001
		Low	22.1/20.6	31/31	<0.0001
CLR	Shannon	High	3.50/3.33	21/23	0.3749
		Medium	3.57/3.44	17/19	0.3749
		Low	3.37/3.36	19/19	0.3749
	Chao1	High	44.6/38.0	59/64	0.4818
		Medium	44.8/40.82	53/61	0.4818
		Low	37.0/37.1	58/63	0.4818
	Pielou	High	0.98/0.98	2/1	0.1722
		Medium	0.98/0.98	1/1	0.1722
		Low	0.98/0.98	1/1	0.1722
	Aitchison	High	39.5/35.1	24/24	0.0004
		Medium	38.60/35.0	24/27	0.0004
		Low	38.1/36.5	25/25	0.0004

^*a*^ CV = coefficient of variation.

**TABLE 2:** Pipelines containing the ASV sequence aggregation method resulted in significantly higher diversity values for all rarefied data regardless of the sequencing depth

Diversity index	Sequencing depth	Mean, ASV/OTU	CV, ASV/OTU	*P* value, mean contrast (ASV vs OTU)
Shannon	10,000	1.53/1.28	48/54	<0.0001
	30,000	1.53/1.28	47/54	<0.0001
	50,000	1.53/1.29	47/54	<0.0001
Chao1	10,000	40.33/36.81	59/65	0.0403
	30,000	41.50/37.96	58/63	0.0403
	50,000	41.91/38.31	57/63	0.0403
Pielou	10,000	0.43/0.37	39/45	<0.0001
	30,000	0.42/0.36	39/46	<0.0001
	50,000	0.42/0.36	39/46	<0.0001
Aitchison	10,000	18.86/16.92	26/28	<0.0001
	30,000	23.88/21.46	26/27	<0.0001
	50,000	26.30/23.70	26/27	<0.0001

**TABLE 3:** Taxonomic outcomes from each bioinformatic pipeline

Density	Bioinformatic pipeline			No. genera	No. genera by relative abundance			Microbiota size (no. bacteria)	% Unassigned genera	Sparsity (% zeros)
	Sequence aggregation	Normalization	Sequencing depth		<0.1%	0.1% to 1%	>1%			
High	OTU	CLR	None	238	NA			476	30	NA
		Rarefaction	Ten	189	137	34	18	465	25	86
			Thirty	192	139	35	18	476	26	86
			Fifty	191	139	34	18	475	26	86
High	ASV	CLR	None	225	NA			712	31	NA
		Rarefaction	Ten	169	114	37	18	701	27	86
			Thirty	172	118	36	18	708	26	86
			Fifty	172	118	36	18	709	26	86
Medium	OTU	CLR	None	238	NA			515	30	NA
		Rarefaction	Ten	207	158	37	12	508	28	86
			Thirty	209	160	37	12	514	28	86
			Fifty	208	159	37	12	513	28	86
Medium	ASV	CLR	None	225	NA			762	31	NA
		Rarefaction	Ten	189	141	36	12	746	28	87
			Thirty	192	144	36	12	757	28	87
			Fifty	192	144	36	12	759	28	87
Low	OTU	CLR	None	238	NA			524	30	NA
		Rarefaction	Ten	218	172	33	13	516	29	87
			Thirty	219	173	33	13	522	29	87
			Fifty	219	173	33	13	522	29	87
Low	ASV	CLR	None	225	NA			721	30	NA
		Rarefaction	Ten	199	154	32	13	713	30	88
			Thirty	199	153	33	13	717	30	87
			Fifty	199	153	33	13	719	30	87

**TABLE 4:** The number of non-overlapping genera did not change significantly between the ASV and OTU sequence aggregation methods by sample density and sequencing depth^*a*^

Sample density	Sequencing depth (××)	No. non-overlapping genera, ASV vs OTU
High	10,000	25
High	30,000	26
High	50,000	26
Medium	10,000	25
Medium	30,000	24
Medium	50,000	25
Low	10,000	23
Low	30,000	24
Low	50,000	24

^*a*^ Density, *P* = 0.21; depth, *P* = 0.37.

There has been considerable debate about the merits of the OTU and ASV methods, and there have been reported differences in taxonomic and ecological outcomes between methods (46). Rather than grouping sequences using an identity threshold like the OTU method, the ASV method treats each sequence as a potential species and can thus resolve sequences within a single nucleotide variation (29). However, the ASV method can inflate the number of taxonomic units generated from 16S rRNA sequences, because it assumes that each bacteria has one copy (47). Many bacteria can have more than one copy of the 16S rRNA sequence, sometimes ranging up to 15 copies (48). The ASV method thus treats each copy as an individual taxonomic unit, while the OTU method groups all copies as one taxonomic unit. In our study, the ASV method generated more taxonomic units, significantly greater alpha and beta diversity values (*P* < 0.05), and larger microbiota sizes (no. bacteria) than the OTU method (Tables 1 and 3).

However, the ASV method did not improve taxonomic classification. Notably, the OTU method generated an equal or slightly lower percentage of unassigned genera than the ASV method (Table 3). The ASV and OTU methods also had some differing taxonomic assignments for low-abundance genera. Our study did not correct for copy numbers and this could have caused the inflated alpha and beta diversity values from the ASV method (49). While several methods exist for copy number correction in 16S rRNA sequence analysis, there is a lack of consensus about the efficacy of existing tools and whether it is necessary for the OTU method (50). By reducing the microbiota size (no. bacteria), the OTU method may have increased the specificity of taxonomic classification at the genus level. This increased specificity of the OTU method could improve the ability to correctly identify microorganisms, thus aiding in the monitoring of spoilage and potentially pathogenic bacteria in food processing environments.

In addition, there are a number of other limitations regarding the use of 16S rRNA amplicon sequencing for characterizing microbiota on the surfaces of food processing environments. Amplicon sequencing of the 16S rRNA gene lacks the resolution to obtain species-level characterization. The process also does not indicate whether the identified taxa are living or dead. Notably, 16S rRNA amplicon sequencing has been primarily used by researchers rather than the food industry for environmental monitoring. However, 16S rRNA amplicon sequencing and other next generation sequencing (NGS) technologies can identify novel taxonomic and ecological characteristics of microbiota collected from surfaces in food processing environments (51). These NGS technologies have the potential to provide greater insight into the influence of the food processing environments on microbiota characteristics, thereby increasing insight into the sources and mechanisms of cross-contamination (41, 52, 53).

### Normalization method can skew diversity and taxonomic outcomes because the environmental surface samples contain a large proportion of low-abundance genera

**Fig 5:**
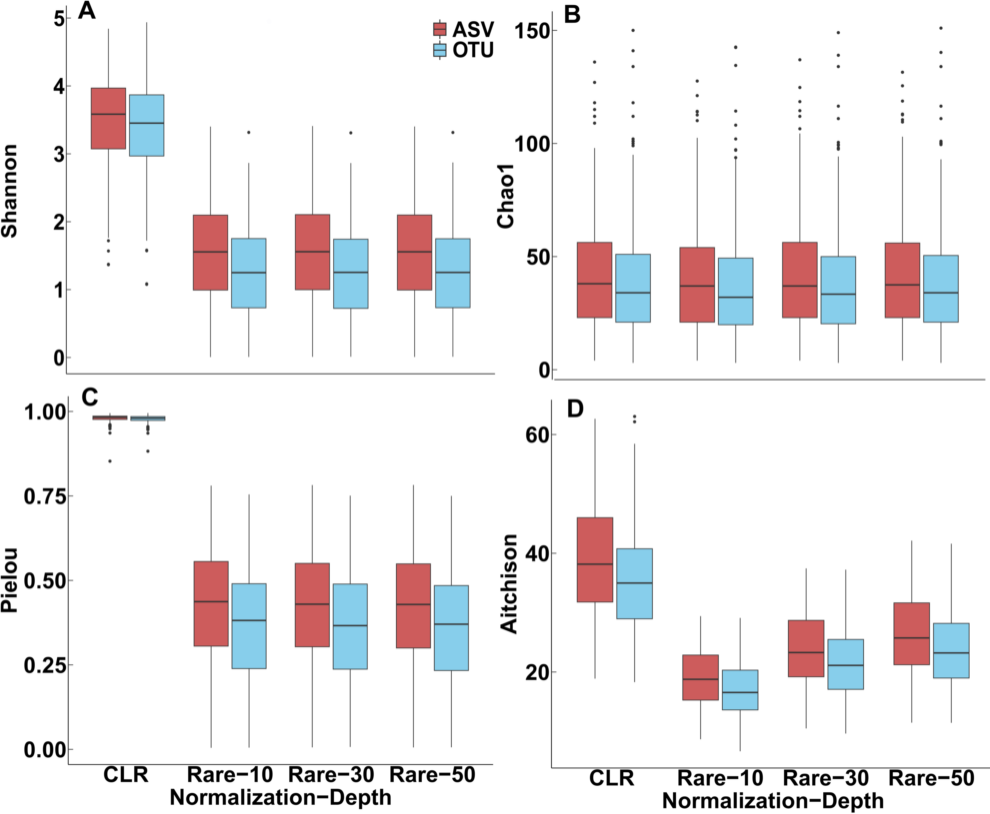
Boxplots of the mean alpha and beta diversity for the (A) Shannon, (B) Chao1, (C) Pielou, and (D) Aitchison indices. The mean alpha and beta diversity values from pipelines containing the ASV method were consistently higher than those calculated from pipelines containing the OTU method. The mean alpha and beta diversity values from pipelines containing the CLR-transformation analysis were consistently higher than pipelines containing rarefaction. Rare-10 = rarefied data-sequencing depth 10,000, Rare-30 = rarefied data-sequencing depth 30,000, Rare-50 = rarefied data-sequencing depth 50,000, and CLR = centered log-ratio transformed data.

Mean alpha and beta diversity values were numerically higher after CLR transformation than for rarefaction (Table 1; Fig. 5). CLR transformation resulted in mean Pielou values equaling 0.98, which is almost exactly even (Table 1). For rarefied samples, the mean Pielou diversity values ranged from 0.40 to 0.46 (Table 1), which is a low level of evenness. Furthermore, CLR transformation decreased the impact of the differences between the ASV or OTU analyses on alpha and beta diversity values. There were no significant differences observed for Shannon, Pielou, and Chao1 diversities for CLR-transformed data (Table 1). CLR transformation resulted in larger microbiota sizes (no. bacteria) and unique genera identified. However, as sequencing depth increased, the number of unique genera and microbiota sizes (no. bacteria) also increased and approached CLR-transformed outcomes (Table 3).

**Fig 6:**
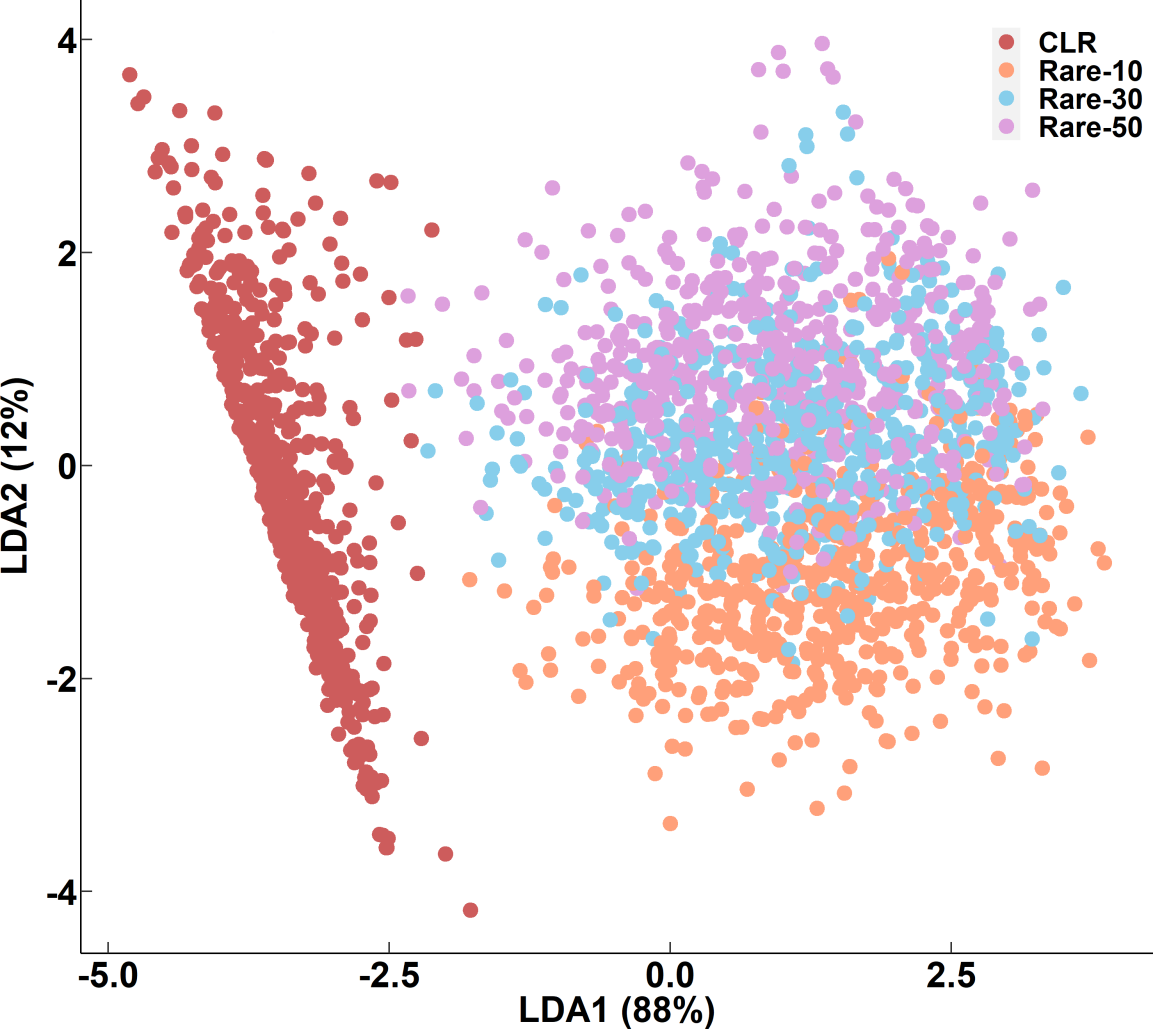
LDA plot of the first two linear discriminants of alpha and beta diversity values. Colors represent normalization methods. Alpha and beta diversity values were able to be separated based on normalization methods.

The pseudo-counts added to the ASV and OTU tables during CLR transformation inflated alpha and beta diversity values and microbiota size (no. bacteria). Our microbial data sets had high degrees of sparsity (>86%) (Table 3) and unevenness, so adding pseudo-counts from CLR transformation increased the “weight” of low-abundance taxa in a data set and decreased the “weight” of high abundance taxa, which inflated alpha and beta diversity values (Fig. 6). Since the average Chao1 diversity values only minimally increased between rarefaction and CLR transformation, the inflation of Shannon, Pielou, and Aitchinson values was mainly due to changes in sample evenness. Also, CLR transformation did not necessarily improve taxonomic assignment since CLR transformation resulted in higher percentages of unassigned genera. Therefore, alpha diversity indices that do not account for evenness in the data set, such as Chao1, might provide better insight into ecological characteristics of uneven or sparse data sets after CLR transformation.

As demonstrated in this study, sequence analysis selection can affect the interpretation of sequencing data derived from the microbiota found on surfaces in dairy processing environments. Normalization method selection should therefore be informed by the expected degree of sparsity and the proportion of low-abundance taxa. While rarefaction has been criticized for being statistically invalid because it drops samples below the minimum library size (54), rarefaction may be better at preserving sample attributes that could be lost through other normalization techniques, particularly for uneven data sets (55). Normalizing samples with uneven microbial composition using CLR transformation may obscure the temporal or spatial variations in the diversity results (8, 9, 41). For example, diversity characterizations of fecal data were optimized when transformed with pseudo counts, while the diversity characterizations of oral microbiomes were optimized when untransformed (56). Furthermore, if rarefaction is selected as the normalization method, then sequencing depth selection is also an important consideration for low-density samples. If sequencing depth is too low, low-abundance taxa may be excluded, but if sequencing depth is higher than most of the library read lengths, too many samples may be lost and low-abundance taxa may be over-emphasized in their taxonomic profile (4). In this study, the average Chao1, Shannon, and Aitchison diversity values and the number of genera increased numerically with increasing sequencing depth (Tables 2 and 3). While rarefaction had some non-significant effects on diversity values, the impact was not as large as the CLR transformation (Fig. 6). In this study, rarefaction better retained the inherent microbial community structure of the surface microbiota.

Microbial community attributes are a vital, but often overlooked, consideration for sequence analysis selection. For example, the 2007 FAMeS (Fidelity of Analysis of Metagenomic Samples) data sets classified simulated metagenomes with a few dominant species as “medium-complexity,” no dominant species as “high-complexity,” and one dominant species as “low-complexity” (57). Their study linked metagenome complexity to bioinformatic outcomes. The samples from our study would be considered “medium-complexity” and demonstrated considerable variability in taxonomic and ecological outcomes. Other microbial community attributes, including dimensionality, sparsity, and flexibility, should be better characterized and connected to bioinformatic analysis selection. In our study, the differences among sequence analyses increased when analyzing low-density samples and low-abundance taxa. Most likely, the unevenness of the environmental surface microbiota contributed to these differences between sequence analysis performances. The environmental surface samples had high degrees of sparsity, though this was influenced somewhat by sample density. Specifically, low-density samples had a higher percentage of unassigned genera, greater unevenness, and greater sparsity than higher-density samples (Tables 1 and 3). Selection of bioinformatic analyses should consider the anticipated structure of the data set to obtain reliable insight into taxonomic and ecological outcomes.

### Conclusion

This study found that different bioinformatic analyses significantly impacted taxonomic and ecological outcomes from low-abundance environmental surface microbiota. The taxonomic characterizations of predominant genera identified on surfaces including *Streptococcus*, *Lactococcus*, *Pseudomonas*, and *Acinetobacter* were relatively unaffected by sequence analysis choice. However, the relative abundances of lower abundance taxa, including *Listeria*, were highly affected by sequence analysis selection. In particular, the ASV aggregation method and normalization using CLR transformation significantly affected the Shannon and Pielou diversity analysis outcomes. We recommend that for sparse, uneven, low-density data sets, the OTU method for sequence aggregation and rarefaction as a normalization method are better for taxonomic and ecological characterization. Further examination of the impact of different bioinformatic analyses on different microbiota attributes should be conducted. Bioinformatic analyses should be rigorously assessed for a variety of microbial data sets and taxonomic and ecological metrics before generic recommendations are made for 16S rRNA sequence analysis.

## Data Availability

The data that support the findings of this study are available in the supplemental material of this article. The sequencing data from this study are openly available at the BioProject accession number PRJNA1075764. The software code from this study is openly available in the GitHub repository at https://github.com/dalysarah/The-choice-of-16S-rRNA-sequence-analysis-impacted-characterization-of-surface-microbiota.

## SUPPLEMENTAL MATERIAL

Table S1 msystems.00620-24-s0001.xlsx Taxonomic and ecological output for each swab sample.

Table S2 msystems.00620-24-s0002.xlsx Object classification.

Table S3 msystems.00620-24-s0003.xlsx Room categories.

Table S4 msystems.00620-24-s0004.xlsx Outersect table.

Table S5 msystems.00620-24-s0005.xlsx Outersect table of low-abundance taxa.

Table S6 msystems.00620-24-s0006.xlsx Paired *t* tests.

ASM does not own the copyrights to Supplemental Material that may be linked to, or accessed through, an article. The authors have granted ASM a non-exclusive, world-wide license to publish the Supplemental Material files. Please contact the corresponding author directly for reuse.

## References

[1] De Oliveira Mota J, Boué G, Prévost H, Maillet A, Jaffres E, Maignien T, Arnich N, Sanaa M, Federighi M. 2021. Environmental monitoring program to support food microbiological safety and quality in food industries: a scoping review of the research and guidelines. Food Control 130:108283. doi:10.1016/j.foodcont.2021.108283

[2] Feng J, Daeschel D, Dooley D, Griffiths E, Allard M, Timme R, Chen Y, Snyder AB. 2023. A schema for digitized surface swab site metadata in open-source DNA sequence databases. mSystems 8:e0128422. doi:10.1128/msystems.01284-22 36847566 PMC10134794

[3] Riesenfeld CS, Schloss PD, Handelsman J. 2004. Metagenomics: genomic analysis of microbial communities. Annu Rev Genet 38:525–552. doi:10.1146/annurev.genet.38.072902.091216 15568985

[4] Franzosa EA, Hsu T, Sirota-Madi A, Shafquat A, Abu-Ali G, Morgan XC, Huttenhower C. 2015. Sequencing and beyond: integrating molecular 'omics' for microbial community profiling. Nat Rev Microbiol 13:360–372. doi:10.1038/nrmicro3451 25915636 PMC4800835

[5] De Filippis F, Valentino V, Alvarez-Ordóñez A, Cotter PD, Ercolini D. 2021. Environmental microbiome mapping as a strategy to improve quality and safety in the food industry. Curr Opin Food Sci 38:168–176. doi:10.1016/j.cofs.2020.11.012

[6] Tan X, Chung T, Chen Y, Macarisin D, LaBorde L, Kovac J. 2019. The occurrence of Listeria monocytogenes is associated with built environment microbiota in three tree fruit processing facilities. Microbiome 7:115. doi:10.1186/s40168-019-0726-2 31431193 PMC6702733

[7] Rolon ML, Tan X, Chung T, Gonzalez-Escalona N, Chen Y, Macarisin D, LaBorde LF, Kovac J. 2023. The composition of environmental microbiota in three tree fruit packing facilities changed over seasons and contained taxa indicative of L. monocytogenes contamination. Microbiome 11:128. doi:10.1186/s40168-023-01544-8 37271802 PMC10240739

[8] Johnson J, Curtin C, Waite-Cusic J. 2021. The cheese production facility microbiome exhibits temporal and spatial variability. Front Microbiol 12:644828. doi:10.3389/fmicb.2021.644828 33767682 PMC7985343

[9] Bokulich NA, Mills DA. 2013. Facility-specific “house” microbiome drives microbial landscapes of artisan cheesemaking plants. Appl Environ Microbiol 79:5214–5223. doi:10.1128/AEM.00934-13 23793641 PMC3753952

[10] Falardeau J, Keeney K, Trmčić A, Kitts D, Wang S. 2019. Farm-to-fork profiling of bacterial communities associated with an artisan cheese production facility. Food Microbiol 83:48–58. doi:10.1016/j.fm.2019.04.002 31202418

[11] Wang S, Wu Q, Nie Y, Wu J, Xu Y. 2019. Construction of synthetic microbiota for reproducible flavor compound metabolism in Chinese light-aroma-type liquor produced by solid-state fermentation. Appl Environ Microbiol 85:e03090-18. doi:10.1128/AEM.03090-18 30850432 PMC6498162

[12] Gu G, Ottesen A, Bolten S, Wang L, Luo Y, Rideout S, Lyu S, Nou X. 2019. Impact of routine sanitation on the microbiomes in a fresh produce processing facility. Int J Food Microbiol 294:31–41. doi:10.1016/j.ijfoodmicro.2019.02.002 30753997

[13] Schön K, Schornsteiner E, Dzieciol M, Wagner M, Müller M, Schmitz-Esser S. 2016. Microbial communities in dairy processing environment floor-drains are dominated by product-associated bacteria and yeasts. Food Control 70:210–215. doi:10.1016/j.foodcont.2016.05.057

[14] Gu G, Ottesen A, Bolten S, Luo Y, Rideout S, Nou X. 2020. Microbiome convergence following sanitizer treatment and identification of sanitizer resistant species from spinach and lettuce rinse water. Int J Food Microbiol 318:108458. doi:10.1016/j.ijfoodmicro.2019.108458 31816526

[15] Quijada NM, Mann E, Wagner M, Rodríguez-Lázaro D, Hernández M, Schmitz-Esser S. 2018. Autochthonous facility-specific microbiota dominates washed-rind Austrian hard cheese surfaces and its production environment. Int J Food Microbiol 267:54–61. doi:10.1016/j.ijfoodmicro.2017.12.025 29291459

[16] Bridier A, Le Grandois P, Moreau MH, Prénom C, Le Roux A, Feurer C, Soumet C. 2019. Impact of cleaning and disinfection procedures on microbial ecology and Salmonella antimicrobial resistance in a pig slaughterhouse. Sci Rep 9:12947. doi:10.1038/s41598-019-49464-8 31506516 PMC6736965

[17] Scholz MB, Lo CC, Chain PSG. 2012. Next generation sequencing and bioinformatic bottlenecks: the current state of metagenomic data analysis. Curr Opin Biotechnol 23:9–15. doi:10.1016/j.copbio.2011.11.013 22154470

[18] Siegwald L, Touzet H, Lemoine Y, Hot D, Audebert C, Caboche S. 2017. Assessment of common and emerging bioinformatics pipelines for targeted metagenomics. PLoS One 12:e0169563. doi:10.1371/journal.pone.0169563 28052134 PMC5215245

[19] Luscombe NM, Greenbaum D, Gerstein M. 2001. What is bioinformatics? A proposed definition and overview of the field. Methods Inf Med 40:346–358. 11552348

[20] Lima J, Manning T, Rutherford KM, Baima ET, Dewhurst RJ, Walsh P, Roehe R. 2021. Taxonomic annotation of 16S rRNA sequences of pig intestinal samples using MG-RAST and QIIME2 generated different microbiota compositions. J Microbiol Methods 186:106235. doi:10.1016/j.mimet.2021.106235 33974954

[21] López-García A, Pineda-Quiroga C, Atxaerandio R, Pérez A, Hernández I, García-Rodríguez A, González-Recio O. 2018. Comparison of mothur and QIIME for the analysis of rumen microbiota composition based on 16S rRNA amplicon sequences. Front Microbiol 9:3010. doi:10.3389/fmicb.2018.03010 30619117 PMC6300507

[22] Walsh AM, Crispie F, O’Sullivan O, Finnegan L, Claesson MJ, Cotter PD. 2018. Species classifier choice is a key consideration when analysing low-complexity food microbiome data. Microbiome 6:50. doi:10.1186/s40168-018-0437-0 29554948 PMC5859664

[23] Prodan A, Tremaroli V, Brolin H, Zwinderman AH, Nieuwdorp M, Levin E. 2020. Comparing bioinformatic pipelines for microbial 16S rRNA amplicon sequencing. PLoS One 15:e0227434. doi:10.1371/journal.pone.0227434 31945086 PMC6964864

[24] Jeske JT, Gallert C. 2022. Microbiome analysis via OTU and ASV-based pipelines-A comparative interpretation of ecological data in WWTP systems. Bioeng (Basel) 9:146. doi:10.3390/bioengineering9040146 PMC9029325 35447706

[25] Cholet F, Lisik A, Agogué H, Ijaz UZ, Pineau P, Lachaussée N, Smith CJ. 2022. Ecological observations based on functional gene sequencing are sensitive to the amplicon processing method. mSphere 7:e0032422. doi:10.1128/msphere.00324-22 35938727 PMC9429940

[26] Chiarello M, McCauley M, Villéger S, Jackson CR. 2022. Ranking the biases: the choice of OTUs vs. ASVs in 16S rRNA amplicon data analysis has stronger effects on diversity measures than rarefaction and OTU identity threshold. PLoS One 17:e0264443. doi:10.1371/journal.pone.0264443 35202411 PMC8870492

[27] Illumina. 2017. 16S metagenomic sequencing library preparation. San Diego, CA.

[28] Rognes T, Flouri T, Nichols B, Quince C, Mahé F. 2016. VSEARCH: a versatile open source tool for metagenomics. PeerJ 4:e2584. doi:10.7717/peerj.2584 27781170 PMC5075697

[29] Callahan BJ, McMurdie PJ, Rosen MJ, Han AW, Johnson AJA, Holmes SP. 2016. DADA2: high-resolution sample inference from Illumina amplicon data. Nat Methods 13:581–583. doi:10.1038/nmeth.3869 27214047 PMC4927377

[30] DeSantis TZ, Hugenholtz P, Larsen N, Rojas M, Brodie EL, Keller K, Huber T, Dalevi D, Hu P, Andersen GL. 2006. Greengenes, a chimera-checked 16S rRNA gene database and workbench compatible with ARB. Appl Environ Microbiol 72:5069–5072. doi:10.1128/AEM.03006-05 16820507 PMC1489311

[31] McMurdie PJ, Holmes S. 2013. Phyloseq: an R package for reproducible interactive analysis and graphics of microbiome census data. PLoS One 8:e61217. doi:10.1371/journal.pone.0061217 23630581 PMC3632530

[32] R Studio Team. 2022. RStudio: integrated development environment for R. 4.2.0. Boston, MA.

[33] Shetty S, Lahti L. 2022. microbiomeutilities: microbiomeutilities: utilities for microbiome analytics. R Packag version 26-4. https://microsud.github.io/microbiomeutilities.

[34] Oksanen J, Simpson G, Blanchet F, Kindt R, Legendre P, Minchin P, O’Hara R, Solymos P, Stevens M, Szoecs E, et al.. 2022. vegan: community ecology package. R Packag version 26-4. https://cran.r-project.org/package=vegan.

[35] Shetty S, Lahti L. 2019. Microbiome R package. R Packag version 26-4

[36] Lenth RV. 2016. Least-squares means: the R package lsmeans. J Stat Softw 69:1–33. doi:10.18637/jss.v069.i01

[37] Fox J, Weisberg S. 2019. An R companion to applied regression. Thousand Oaks, CA Sage. https://www.john-fox.ca/Companion/.

[38] Venables WN, Ripley BD. 2002. Modern applied statistics with S. Springer. Available from: www.stats.ox.ac.uk/pub/MASS4/VR4stat.pdf

[39] Stobnicka-Kupiec A, Gołofit-Szymczak M, Górny R. 2019. Microbial contamination level and microbial diversity of occupational environment in commercial and traditional dairy plants. Ann Agric Environ Med 26:555–565. doi:10.26444/aaem/112381 31885228

[40] Stellato G, De Filippis F, La Storia A, Ercolini D. 2015. Coexistence of lactic acid bacteria and potential spoilage microbiota in a dairy processing environment. Appl Environ Microbiol 81:7893–7904. doi:10.1128/AEM.02294-15 26341209 PMC4616952

[41] Stellato G, La Storia A, De Filippis F, Borriello G, Villani F, Ercolini D. 2016. Overlap of spoilage-associated microbiota between meat and the meat processing environment in small-scale and large-scale retail distributions. Appl Environ Microbiol 82:4045–4054. doi:10.1128/AEM.00793-16 27129965 PMC4907188

[42] Kable ME, Srisengfa Y, Xue Z, Coates LC, Marco ML. 2019. Viable and total bacterial populations undergo equipment- and time-dependent shifts during milk processing. Appl Environ Microbiol 85:1–14. doi:10.1128/AEM.00270-19 PMC6581186 31028031

[43] Caraballo Guzmán A, González Hurtado MI, Cuesta-Astroz Y, Torres G. 2020. Metagenomic characterization of bacterial biofilm in four food processing plants in Colombia. Braz J Microbiol 51:1259–1267. doi:10.1007/s42770-020-00260-x 32221908 PMC7455661

[44] de Cena JA, Zhang J, Deng D, Damé-Teixeira N, Do T. 2021. Low-abundant microorganisms: the human microbiome’s dark matter, a scoping review. Front Cell Infect Microbiol 11:689197. doi:10.3389/fcimb.2021.689197 34136418 PMC8201079

[45] Melo J, Andrew PW, Faleiro ML. 2015. Listeria monocytogenes in cheese and the dairy environment remains a food safety challenge: the role of stress responses. Food Res Int 67:75–90. doi:10.1016/j.foodres.2014.10.031

[46] Joos L, Beirinckx S, Haegeman A, Debode J, Vandecasteele B, Baeyen S, Goormachtig S, Clement L, De Tender C. 2020. Daring to be differential: metabarcoding analysis of soil and plant-related microbial communities using amplicon sequence variants and operational taxonomical units. BMC Genomics 21:733. doi:10.1186/s12864-020-07126-4 33092529 PMC7579973

[47] Schloss PD. 2021. Amplicon sequence variants artificially split bacterial genomes into separate clusters. mSphere 6:e0019121. doi:10.1128/mSphere.00191-21 34287003 PMC8386465

[48] Klappenbach JA, Saxman PR, Cole JR, Schmidt TM. 2001. Rrndb: the ribosomal RNA operon copy number database. Nucleic Acids Res 29:181–184. doi:10.1093/nar/29.1.181 11125085 PMC29826

[49] Kembel SW, Wu M, Eisen JA, Green JL. 2012. Incorporating 16S gene copy number information improves estimates of microbial diversity and abundance. PLoS Comput Biol 8:e1002743. doi:10.1371/journal.pcbi.1002743 23133348 PMC3486904

[50] Louca S, Doebeli M, Parfrey LW. 2018. Correcting for 16S rRNA gene copy numbers in microbiome surveys remains an unsolved problem. Microbiome 6:41. doi:10.1186/s40168-018-0420-9 29482646 PMC5828423

[51] Yap M, Ercolini D, Álvarez-Ordóñez A, O’Toole PW, O’Sullivan O, Cotter PD. 2022. Next-generation food research: use of meta-omic approaches for characterizing microbial communities along the food chain. Annu Rev Food Sci Technol 13:361–384. doi:10.1146/annurev-food-052720-010751 34678075

[52] Zwirzitz B, Wetzels SU, Dixon ED, Stessl B, Zaiser A, Rabanser I, Thalguter S, Pinior B, Roch FF, Strachan C, Zanghellini J, Dzieciol M, Wagner M, Selberherr E. 2020. The sources and transmission routes of microbial populations throughout a meat processing facility. NPJ Biofilms Microbiomes 6:26. doi:10.1038/s41522-020-0136-z 32651393 PMC7351959

[53] Lacorte GA, Cruvinel LA, de Paula Ávila M, Dias MF, de Abreu Pereira A, Nascimento AMA, de Melo Franco BDG. 2022. Investigating the influence of food safety management systems (FSMS) on microbial diversity of Canastra cheeses and their processing environments. Food Microbiol 105:104023. doi:10.1016/j.fm.2022.104023 35473976

[54] McMurdie PJ, Holmes S. 2014. Waste not, want not: why rarefying microbiome data is inadmissible. PLoS Comput Biol 10:e1003531. doi:10.1371/journal.pcbi.1003531 24699258 PMC3974642

[55] Schloss PD. 2024. Waste not, want not: revisiting the analysis that called into question the practice of rarefaction. mSphere 9:e0035523. doi:10.1128/msphere.00355-23 38054712 PMC10826360

[56] Thorsen J, Brejnrod A, Mortensen M, Rasmussen MA, Stokholm J, Al-Soud WA, Sørensen S, Bisgaard H, Waage J. 2016. Large-scale benchmarking reveals false discoveries and count transformation sensitivity in 16S rRNA gene amplicon data analysis methods used in microbiome studies. Microbiome 4:62. doi:10.1186/s40168-016-0208-8 27884206 PMC5123278

[57] Mavromatis K, Ivanova N, Barry K, Shapiro H, Goltsman E, McHardy AC, Rigoutsos I, Salamov A, Korzeniewski F, Land M, Lapidus A, Grigoriev I, Richardson P, Hugenholtz P, Kyrpides NC. 2007. Use of simulated data sets to evaluate the fidelity of metagenomic processing methods. Nat Methods 4:495–500. doi:10.1038/nmeth1043 17468765

